# Core health system measures response to COVID-19 among East Asian countries

**DOI:** 10.3389/fpubh.2024.1385291

**Published:** 2024-06-03

**Authors:** Jun Jiao, Wei Chen

**Affiliations:** ^1^School of Population and Health, Renmin University of China, Beijing, China; ^2^Yichun Hospital of Traditional Chinese Medicine, Yichun, Jiangxi, China

**Keywords:** COVID-19, core health system, containment strategy, mitigation strategy, East Asia

## Abstract

**Objective:**

The purpose of this study is to summarize the health system response to COVID-19 in four East Asian countries, analyze the effectiveness of their health system response, and provide lessons for other countries to control the epidemic and optimize their health system response.

**Methods:**

This study investigated and summarized COVID-19 data and health system response in four East Asian countries, China, Japan, Mongolia, and South Korea from national governments and ministries of health, WHO country offices, and official websites of international organizations, to assess the effectiveness of health system measures.

**Result:**

As of June 30, 2022, all four countries are in a declining portion of COVID-19. China has two waves, and new cases increased slowly, with the total cases per million remaining within 4, indicating a low level. Japan has experienced six waves, with case growth at an all-time high, total cases per million of 250.994. Mongolia started the epidemic later, but also experienced four waves, with total cases per million of 632.658, the highest of the four countries. South Korea has seen an increasing number of new cases per wave, with a total case per million of 473.759.

**Conclusion:**

In containment strategies adopted by China and Mongolia, and mitigation strategies adopted by Japan and South Korea, health systems have played important roles in COVID-19 prevention and control. While promoting vaccination, countries should pay attention to non-pharmaceutical health system measures, as evidenced by: focusing on public information campaigns to lead public minds; strengthening detection capabilities for early detection and identification; using technical ways to participate in contact tracing, and promoting precise judging isolation.

## 1 Introduction

COVID-19 pandemic declared by the World Health Organization (WHO) in March 2020 has had far-reaching effects on people’s lives, health systems and wider society (1). As of June 30, 2022, the global cumulative number of confirmed cases exceeded 546.1 million for 546,100,990 cases and 6,333,828 deaths. Based on the United Nations Population Fund statistics of the global population, there are approximately 7,953.95 million people in 2022. With a mortality rate of over 1%, the average global diagnosis rate is 1 in 15 persons (2). Of these, a total of 258,324,223 cases have been diagnosed so far in 2022 alone, accounting for 47% of the cumulative new cases and 57% of the cumulative deaths in 2021 alone. On November 24, 2021, South Africa reported to the WHO the discovery of the omicron variant, in which infection speed and immune escape are very high (3, 4). Affected by the omicron variant, the global fourth wave began in 2022, and a new wave of COVID-19 began in various countries.

Since the first reported COVID-19 cases, countries have responded differently at different times and with different health system responses. China, Japan, Mongolia, and South Korea among East Asian countries were selected for this paper, and North Korea was not included in this study because the first COVID-19 case was reported on May 12, 2022. China is an upper-middle-income country, and effort to expand disease coverage in coming years adopted containment strategies with a strict blockade and proactive case management, makes full use of the unique advantages of traditional Chinese medicine. Japan and South Korea are high-income countries, generally have the highest spending on health care and the average percentage spending on health care is 5.5% of total GDP spent, adopted mitigation strategies to flatten the epidemic curve, targeting vulnerable populations and managing the seriously ill (5, 6). Japan is primarily managed by private organizations under universal health coverage (7). South Korea started “Living with the COVID-19” mode on November 1, 2021, and aims to implement universal health coverage; however, cost sharing is generally quite high, resulting in people still relying largely on private insurance. Mongolia, a lower-middle-income country, is on track to achieve universal health coverage by strengthening primary health care, and adopting containment strategies but had some problems with open borders and mitigating preventive and control measures.

Currently, 67.7% of the world population has received at least one dose of COVID-19 vaccines. 60.67% of the global population is fully vaccinated and 5.50% is incompletely vaccinated. China reached 90.75%, Japan reached 83.27%, Mongolia reached 67.90% (as of May 18, 2022) and South Korea reached 86.97% vaccination (8). Based on previous estimates of COVID-19 thresholds ranging from about 60–70%, current vaccination in many countries has exceeded this predicted herd immunity threshold, without taking into account previously confirmed cases (5, 9). Also, as with other seasonal coronaviruses, COVID-19 is capable of reinjecting people who already have the disease, but the frequency of reinfection is not known (10–12).

In this context, while promoting vaccination, we must pay attention to non-pharmacological interventions. The health system, as the main force in COVID-19 prevention and control, the health system undertakes the detection, identification, and treatment of cases; the health system undertakes the epidemiological investigation and management of cases, and so on (13, 14). Second, the ongoing management of the health system response to COVID-19 is not only about disease management but more importantly, about managing people. If not managed well, there will be significant psychological and social impacts that will have long-term effects on individuals and society (15). In addition, health system response is closely related to a country’s COVID-19 mortality rate. Given that COVID-19 is still in a global pandemic situation and poses a risk to people’s life safety and health, and also impacts the world economy. So, research on the health system’s response to COVID-19 is necessary. The purpose of this study is to summarize the health system response to COVID-19 in four East Asian countries, analyze the effectiveness of their health system response, and provide lessons for other countries to control the epidemic and optimize their health system response.

## 2 Methods

### 2.1 Statistic of COVID-19 data

This paper investigated new cases per million and total deaths per million in China, Japan, Mongolia, and South Korea from the first reported cases to June 30, 2022. Data on COVID-19 cases are from Coronavirus Resource Centre at Johns Hopkins University^1^ and WHO Coronavirus Dashboard.^2^ The inclusion criteria for confirmed cases of COVID-19 in China is based on the Chinese Clinical Guidance for COVID-19 Pneumonia Diagnosis and Treatment published by the Chinese Health Commission. Classification of clinical manifestation was added in Hubei Province in February 2020, and it included suspected cases with imaging features of pneumonia to clinical diagnosis cases.

### 2.2 Health system measures in COVID-19

Health system measures were searched from the first reported cases around the world to June 30, 2022. The paper focused on COVID-19 measures used by national health system, thus we searched for health system measures in response to COVID-19 from national governments and ministries of health. Such as China’s Health Committee,^3^ Japan’s Ministry of Health, Labor and Welfare,^4^ Mongolia’s Ministry of Health,^5^ and South Korea’s Ministry of health and welfare.^6^ COVID-19 as a national public health emergency, some measures were directly issued by government departments, so the paper included national official websites in the search, such as China,^7^ Japan (see Footnote 4), Mongolia (see Footnote 5), and South Korea.^8^ The search used the following keywords: COVID-19, policy, response and strategy, and followed the inclusion–exclusion criteria: (1) The health system needs to play a role in measures, (2) Measure has a national scope, (3) Exclude duplication measures. After collecting data and health system measures, we mapped the national epidemic curve over time, sorted out through measures, and listed public information campaigns, testing policy, contact tracing, vaccination, isolation and quarantine policy.

## 3 Results

### 3.1 National measures to prevent and control COVID-19

China, Japan, Mongolia, and South Korea are the four East Asian countries that suffered from COVID-19 earlier and are the representative countries’ responses to COVID-19. Public health measures have played major roles in all COVID-19 stages. China has taken many measures in public health measures to achieve precise prevention and control and universal coverage. On January 1, 2020, a case of pneumonia of unknown cause was reported, and the epidemic information was released daily on the official website. China maintains a good public information campaign while keeping information open. The testing policy has been supported by people from all sectors of society. The testing cost continues to decrease, and the testing facilitation has been gradually promoted, with multiple rounds of testing conducted in cluster epidemics. China is using information technology and big data to contact tracing and isolate close contacts and is accelerating vaccination. In addition, China has issued multiple editions of COVID-19 Diagnosis and Treatment Plan, detailing treatment plans at various stages, not only adopting modern medicine and public health measures, but also traditional Chinese medicine theories and traditional Chinese medicine are widely used to deal with COVID-19.

Japan issued a level 1 risk warning on time, released a short video on COVID-19 prevention, and appealed to the public with “self-restraint.” Unlike in other countries, the decision to test in Japan is mainly made by physicians. Initially, testing was only available for symptomatic individuals, but later it became available for asymptomatic individuals when doctors deemed it necessary, and free Polymerase chain reaction (PCR) and antigen testing have become available for asymptomatic individuals. In contact tracing, Japan has adopted ‘cluster busting,’ which focuses on large tracking, clustered epidemics. On February 17, 2020, Japan officially vaccinated, with priority given to medical workers, the older adult, and those with pre-existing conditions. In addition, Japan advocates home isolation for mild, asymptomatic infections and hospitalization for severe cases.

Mongolia launched a one-month outbreak prevention and control operation in April 2020 and the highest level of national disaster preparedness on high alert in November. Citizens were called to pay attention to COVID-19 through official websites and the distribution of pamphlets. To facilitate testing for the public, testing centers are being established in all cities and specialized hospitals to provide drop-off-free testing services, and One Citizen-One Household is being launched. In March 2022, the traveler testing requirement was eliminated. Contact tracing in Mongolia is done with the help of scanning QR codes in public places, isolation of close contacts, and medical observation of sub-close contacts. On March 23, 2021, Mongolia officially launched vaccination with the same order of priority as Japan and suspended vaccination during the blockade. In response to COVID-19, Mongolia has lockdown several times. At various times, Mongolia requires quarantine for people entering the country from different regions. On March 14, 2022, Mongolia officially lifted its entry quarantine restrictions.

On January 27, 2020, South Korea raised the public alert level to orange and passed COVID-19 Three Act in February. On January 20, 2020,South Korea managed to get testing centers up and running, initially making testing available to suspected cases and patients under investigation, and then gradually liberalizing and simplifying the procedure for COVID-19 testing in March 2022 by recognizing rapid antigen-positive results. In February 2020, close contact tracing software was used to trace people within 100 meters of COVID-19 cases. In February 2021, South Korea officially started universal vaccination. A quarantine policy was adopted for confirmed patients, and inbound travelers were quarantined. After May 2021, travelers who have completed COVID-19 vaccination in South Korea were exempted from quarantine. Table 1 summarizes Core health system measures response to COVID-19 in China, Japan, Mongolia, and South Korea, including public information campaigns, testing policy, contact tracing, vaccination, isolation and quarantine policy.

**Table 1:** Core health system measures response to COVID-19 in China, Japan, Mongolia, and South Korea.

Country Measure	China	Japan	Mongolia	South Korea
Public information campaigns	(1) Starting from December 31, 2019, and January 21, 2020, Wuhan and the National Health Commission published nationwide epidemic information on official websites on a daily basis. (2) Advises residents to avoid enclosed public venues, wear masks and seek medical consultations in time in case of fever and respiratory infection symptoms. (3) On February 1, 2020, The National Health Commission issued the “Guiding Manual for the Prevention of COVID-19 Pandemic.”	(1) On January 21, 2020, Level 1 risk warning were issued. (2) The Ministry of Health, Labor and Welfare issued a Basic policy on COVID-19 Response, and the prime minister called on the public to “exercise self-restraint.” (3) On March 23, 2020, cabinet Office provided comprehensive information on its website. (4) On April 7, 2020, a one-month “state of emergency” order was declared and lifted on May 25. (5) Short movies for the public campaign on Coronavirus.	(1) Mongolia Ministry of Health general public website for coronavirus was launched. (2) Since November 12, 2020, the country has been on high alert for disaster prevention, which is the highest state for disaster prevention in Mongolia. (3) On April 13, 2020, the State Emergency Commission began promoting a month-long campaign. (4) On April 7, 2020, a state of emergency was introduced in Japan, which was extended from March 2 to March 30, 2021.	(1) On January 27, 2020, the government raised the public alert level to orange (level 3 out of 4). (2) On February 26, 2020, the Law on Prevention and Management of Infectious Diseases, Quarantine, and Medical Treatment were adopted. (3) On October 25, 2021, the draft quarantine plan was announced and the mode of “living with COVID-19” began on November 1. (4) In April 2022, COVID-19 was reclassified as a Class B infectious disease and applied to the standards of the general medical system.
Testing policy	(1) Before January 20, 2020, samples had to sent to a laboratory in Beijing for testing. And there are certain criteria for the test. (2) On March 31, 2020, intensify the screening of asymptomatic cases. (3) Ministry of Health issued a notice on April 18, 2020, calling all parts of the country to increase testing capacities and ensure testing accuracy. (4) For cities or regions with clusters of new cases, repeated large-scale tests for all residents have taken place. (5) Multiple rounds to reduce the cost of testing.	(1) Testing policy was revised to target most symptomatic individuals. (2) Government is ramping up capacity to test, and referral by a doctor based on symptoms to be required to receive a test. (3) From March 6, 2021, Medical insurance was applied to PCR tests. (4) On November 12, 2021, the Japanese government announced a change in testing policy. Free PCR and antigen testing have become available for asymptomatic individuals.	(1) On November 13, 2020, Government procured 100,000 COVID-19 Rapid Ag tests. (2) Testing centers established at some cities and all specialized hospitals to perform COVID-19 rapid antigen tests. (3) From December 4 to 11, 2020, Mongolia carried out “One Citizen-One Household” campaign: at least one member of each household was tested. (4) Drive-through COVID-19 testing is available at Interned Hospital in Ulaanbaatar.	(1) At the 50 designated health facilities from February 7, 2020, improved real-time PCR (polymerase chain reaction) can detect Coronavirus within 6 h. (2) From April 3, 2020, all patients classified as suspected cases and Patients Under Investigation may get tested. (3) On March 11, 2022, the South Korean government simplified the testing of COVID-19. Specifically, people who tested positive from rapid antigen tests are considered as positive even without results from PCR tests.
Contact tracing	(1) Close contacts, secondary close contacts, and general contacts were traced. (2) Close contacts were isolated and put under medical observation. (3) Implement closed and grid management of the community, and carry out active tracking and personnel management. (4) Identify close contacts through big data health codes and place codes. And hold a press conference, assign yellow code and other requirements during this period and the patient in the same place in the personnel report.	(1) Close contacts should pay attention to their health conditions for 14 days, and inform a healthcare center before visiting a medical institution if they develop a fever or respiratory symptoms. (2) In cluster epidemic, tracing the links of each infected person, focusing on key areas. (3) Japan has instead used ‘cluster busting’ contact tracing which targets large or super-spreader events. (4) The COVID-19 Contact-Confirming Application and manually conducted was used.	(1) People who came into close contact with the patient would be quarantined, and people with indirect contact would be taken down and under medical observation. (2) Mongolia is contact tracing through scanning QR codes in public places.	(1) The Corona 100 m app launched on February 11 and, using government data, alerts users when they come within 100 m of a location visited by an infected person. (2) Comprehensive contact tracing, with dishonesty criminally punishable. (3) All contacts of cases are required to self-isolate.
Vaccination	(1) In December 2020, the vaccination campaign for key groups was launched. In July 2021, Anhui, Guangxi and Jiangsu provinces launched COVID-19 vaccination for minors. In October 2021, fully activate booster needle. Sequential immunization with booster needles was implemented in February 2022. (2) First, vaccinate some key groups. (3) The specification for nucleic acid 20-in-1 mixed Detection Technique was published.	(1) On December 3, 2020, Japan’s parliament enacted a law to cover the costs for residents to be vaccinated. (2) It would prioritize medical workers, the older adult, and those with pre-existing conditions. (3) On June 17, 2021, inoculation tickets and bookings were opened to 18–64-year-olds to receive their first dose. (4) On September 13, 2021, healthcare workers began the dispatch of “vaccine cars,” to the younger in smaller neighborhoods.	(1) On February 23, 2021, Mongolia launched a nationwide COVID-19 vaccination campaign. (2) It would prioritize healthcare workers at high risk, older people and those with health issues. After that are employees of strategically important sectors, such as border employees and coal-hauling truck drivers and employees of all levels of educational organizations. (3) On April 10, 2021, Vaccination in Mongolia was suspended during the re-lockdown.	(1) The older adult, vulnerable groups, and essential service personnel such as health care professionals will be the first in line to receive the vaccine as they account for about 36 million people. (2) In February 2021, South Korea began offering free vaccinations to all citizens.
Isolation and quarantine policy	(1) Traffic leaving Wuhan will be temporarily closed from 10:00 on January 23, 2020. Citizens were asked not to leave Wuhan and to stay at home. The lockdown of Wuhan lasted 76 days. (2) Wuhan rapidly built 16 cabin hospitals to isolate patients with mild diseases. (3) Close contacts and inbound passengers were quarantined for 14 days, which was later shortened to 7 days.	(1) On February 1, 2020, the decree allowed authorities to require suspected patients to accept quarantines and be hospitalized. (2) Home recuperation for mild or asymptomatic infected persons. Positive patients should be isolated at home for at least 7 days. (3) From June 10, 2022, 98 low-risk countries were allowed to travel without quarantine.	(1) From April 10 to 25, 2020, Mongolia began to lockdown country, which was extended to May 8, and then lockdown several times. (2) In November 2021, to prevent the Omicron variant strain, the entry passengers from 11 countries were quarantined for 10 days. (3) On March 13, 2020, No quarantine is required for all travelers.	(1) Isolating confirmed patients should be adopted. (2) From February 4, 2020, the special entry procedures were gradually extended to all inbound passengers worldwide. (3) Starting April 1, 2020, all inbound passengers should be quarantined for 14 days, and those who violate the rules must wear wristbands. (4) Starting May 5, 2021, people who have received COVID-19 vaccines in South Korea can be exempted from quarantine upon re-entry.

### 3.2 COVID-19 trends

**Figure 1:**
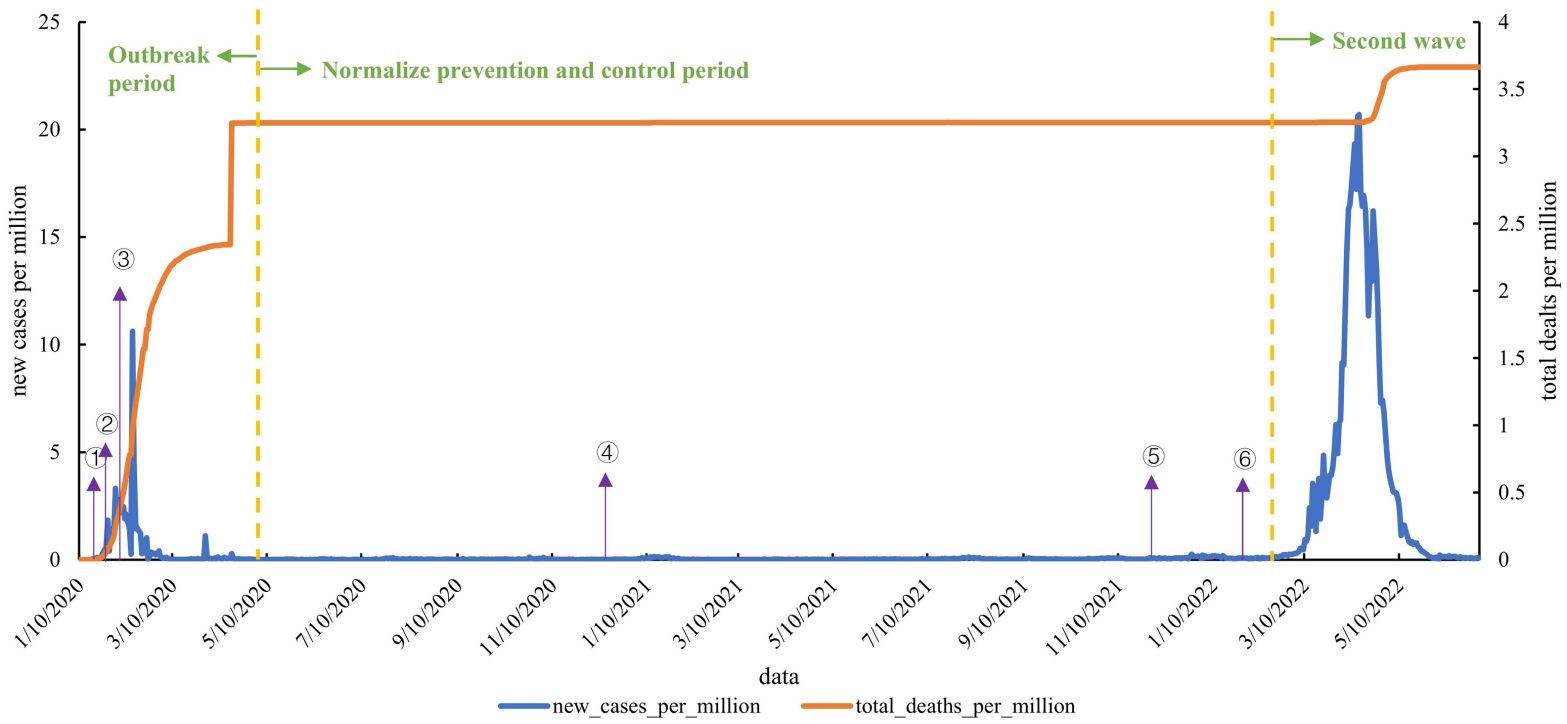
Epidemic curve of local cases of COVID-19 in China. (1) On January 21, 2020, the National Health Commission began issuing national outbreak information. (2) On January 23, 2020, lockdown Wuhan city. (3) On February 1, 2020, The National Health Commission issued the “Guiding Manual for the Prevention of COVID-19 Pandemic.” (4) In December 2020, vaccination of key populations began. (5) On December 7, 2021, the general strategy of “external prevention of importation and internal prevention of rebound” and the general policy of “dynamic clearance” were established. (6) In February 2022, booster vaccination was introduced. In February 2022, the booster vaccine was introduced.

As shown in Figure 1, COVID-19 epidemic in China is divided into 3 periods: the outbreak period, normalized prevention and control period, and the second wave, which was cut off on May 8, 2020, and March 2022. After the first COVID-19 case was reported, China came into the outbreak period, and COVID-19 cases were increasing severely. On February 13, 2020, new cases per million reached 10.613, and then the growth of new cases gradually decreased. After the first death was reported on January 11 and reached a high point after the recount of cases on April 30. During the normalized prevention and control period, there was no nationwide epidemic, but cluster epidemics occurred sometimes. As we entered March 2022, the impact of the fourth global COVID-19 wave, coupled with the omicron characteristics, led to a marked increase in the frequency and scope of the epidemic, with new cases even surpassing the first wave. New cases per million peaked on April 14, 2022, showing a clear crest, while deaths. There is also an increase in the number of deaths.

**Figure 2:**
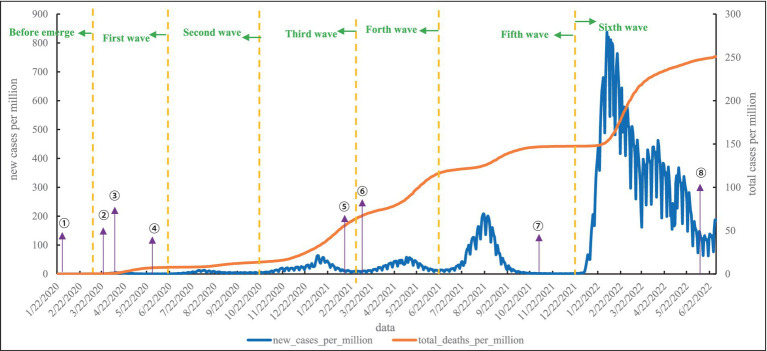
Epidemic curve of local cases of COVID-19 in Japan. (1) On February 1, 2020, the decree allowed authorities to require suspected patients to accept quarantines and be hospitalized. (2) On March 23, 2020, Binet Office provided comprehensive information on its website. (3) From April 7, 2020, a state of emergency was introduced in Japan. (4) On May 25, 2020, the state of emergency was lifted nationwide. (5) On February 17, 2021, Japan began vaccinating medical personnel with the COVID-19 vaccine. (6) From March 6, 2021, Medical insurance was applied to PCR tests. (7) On November 12, 2021, the government announced a change in testing policy, free PCR and antigen testing have become available for asymptomatic individuals. (8) On June 10, 2022, travel was opened to 98 countries with low risk, exempt from quarantine.

As shown in Figure 2, the first COVID-19 case in Japan was reported on January 15, 2020. In February, there was a cluster epidemic on the Diamond Princess cruise ship. The first wave of COVID-19 began in March 2020, when Japan entered a state of emergency after a record-high number of new daily cases due to improved detection capabilities and many imported cases. In the first wave, the mortality rate was much higher than in the other waves, which remained largely above 0.1, and on May 11, the mortality rate exceeded 0.5. After a normalized period, the second wave broke out in June 2020. Entering late October 2020, with lower temperatures, Japan entered the third wave, which peaked on January 15, 2021, with new cases per million reaching 57.562 and total cases per million reaching 35.47. The fourth wave was quite similar to the third wave in terms of cycle and peak. Entering July 2021, the fifth wave dominated by the Delta followed, and in mid-August, the epidemic worsened, with more than 25,000 daily new cases. After entering October, new cases declined rapidly. Entering 2022 as in the other three countries, infections soared due to the omicron, and the speed and scale of infection far surpassed the previous five waves. As of June 30, 2022, the total cases per million in Japan reached 250.994.

**Figure 3:**
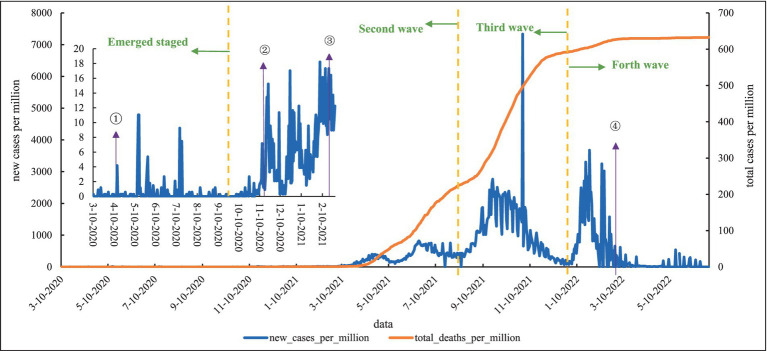
Epidemic curve of local cases of COVID-19 in Mongolia. (1) From April 10 to 25, 2020, Mongolia began to lockdown country, which was extended to May 8, and then lockdown several times. (2) From November 12, 2020, a state of high alert for national disaster preparedness was in effect. (3) From December 4 to 11, 2020, Mongolia carried out “One Citizen-One Household” campaign. (4) On February 23, 2021, Mongolia launched a nationwide COVID-19 vaccination campaign. (5) On March 14, 2022, all travelers do not need pre-travel tests max 72 h before departure.

As shown in Figure 3, the first COVID-19 case was registered by French nationals on 10 March 2020, and subsequently, new cases spread rapidly after the first diagnosis (16). In addition, there were several peak points in the emerging stage. The first death case in Mongolia appeared on December 18, 2020, which was also the second wave. In the second wave, Mongolia was in an epidemic crisis, with new cases constantly showing a backlash tense, with constant ups and downs, and an increase in deaths. Compared to the second wave, the third wave came with a vengeance, and this was the wave with the most rapid increase in deaths. The third wave hits and the Delta spreads, on October 31, 2021, reaching a peak of 7335.006 new cases per million. With the accelerated global spread of omicron and a significant increase in the number of people entering and leaving Mongolia, the fourth wave was opened. Mongolia’s total cases per million are the highest of the four countries. As of June 30, 2022, Mongolia’s total cases per million reached 632.658.

**Figure 4:**
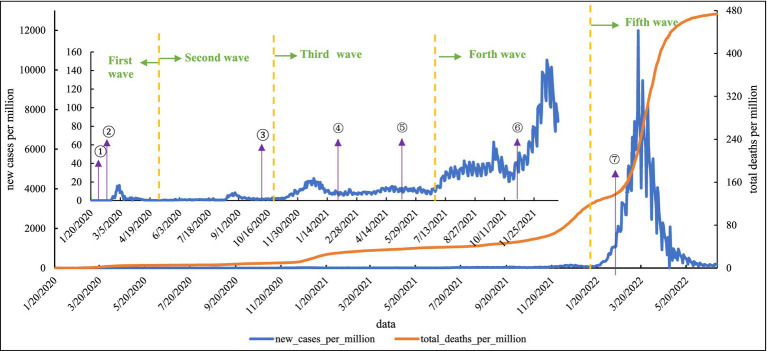
Epidemic curve of local cases of COVID-19 in South Korea. (1) On January 27, 2020, the government raised the public alert level to orange. (2) On February 26, 2020, the Amendment to the Infection Disease Prevention and Management Act, the Amendment to the Quarantine Act, and the Amendment to the Medical Treatment Act, the “COVID-19 Three Acts,” were passed in response to the COVID-19 outbreak. (3) From October 13, 2020, face masks are compulsory in public places. (4) On February 2021, South Korea began free vaccination. (5) From May 5, 2021, people who have received the COVID-19 vaccine within South Korea would be granted exemption from quarantine when re-entering from abroad. (6) On November 1, 2021, the South Korean government started the “Coexistence with Neosporin” model. (7) On March 11, 2022, the South Korean government simplified the testing of COVID-19.

As shown in Figure 4, there are five distinct peaks of COVID-19 in South Korea. The first COVID-19 case was reported on January 20, 2020, and the number of confirmed cases began to increase in February and reached the peak on February 29, which was associated with a religious group. From April to early August, new cases per million remained around 1, but in August, the second wave began in the metropolitan area churches and continued to spread, with frequent clusters in non-metropolitan areas and a nationwide multipoint spread. The interval between the second and third waves was shortened, and new cases rose again to more than 300 in a single day after 81 days since August 29, showing signs of a “third pandemic.” The fourth wave followed, and the deaths increased even more rapidly. On December 17, 2021, new cases per million reached 141.096, and by the 25th, total deaths per million exceeded 100. By 2022, the fifth wave was growing rapidly, with daily new cases per day reaching the highest in the world. At the peak of the fifth wave, on March 16, new cases per million reached 11,987.562, and by June 30, 2022, South Korea’s total cases per million reached 473.759.

## 4 Discussion

This study describes health systems measures used by the four East Asian countries – China, Japan, Mongolia, and South Korea – to respond to COVID-19 and their effectiveness in controlling new cases and deaths. China and Mongolia adopted containment strategies, adhered to a lockdown policy, adopted restrictions on social distance, strict public health measures, and proactive case detection and management. South Korea and Japan adopted mitigation strategies, recommending home isolation for mild cases in an attempt to flatten the policy curve and ease pressure on their healthcare systems (5).

Health system measures in response to COVID-19 include the following two main categories: first, policies to strengthen the health system capacity, such as new square cabin hospitals and coordination of medical resources; and second, policies to reduce people contracting the virus, such as wearing masks and isolating close contacts (17). This paper discusses the experiences and shortcomings of the response to COVID-19 in terms of five health system measures: public information campaigns, testing policy, contact tracing, vaccination, and isolation and quarantine policy.

### 4.1 Public information campaigns

Public information campaigns are the beacon of COVID-19 epidemic prevention and control, and the leader of people’s ideological trends, which are important policy tools used worldwide (18). Public information campaigns aiming at shaping beliefs, attitudes, Social norms, and actual behaviors in (a segment of) the mass public, and play an important role in educating people about COVID-19, promoting the acceptance and compliance of policies and measures, and promoting the implementation of policies (19). Public information campaigns are also considered one of the cost-effective intervention measures, which include promulgating policies and decrees, announcing risk levels and the state of country, releasing COVID-19-related public information, etc. (20).

All four countries have been publishing national epidemic information on their official websites to maintain the authority and reliability of the information. China continues to revise its “Guiding Manual for the Prevention of COVID-19 Pandemic” to inform the public about COVID-19 in various. China ranked first in the public perception survey of 19 countries severely affected by COVID-19, with high trust in government and much higher than average satisfaction with COVID-19 policies (21). The Japanese population exhibited higher self-regulation and health literacy. The suppression of the first COVID-19 wave is thought to be attributed to the increased risk perception of people after receiving information from government and media reports (22). In public information campaigns, people are only called for self-restraint, without coercion, punishment, or compensation. The new cases also maintain a relatively stable level in the early stages of the epidemic (23). Mongolia initially provided infection updates through the Ministry of Health’s daily briefings and currently provides infection updates through an online portal. However, due to its inadequate emergency management capacity and vulnerable health care resources, public confidence in the government’s ability to respond to a pandemic is lacking. Mongolia has repeatedly entered a state of emergency in an attempt to slow the outbreak through a blockade (24). South Korea’s public information campaigns guide the direction of its epidemic prevention, from the adoption of COVID-19 III law in 2020, with a strict contact tracing pathway, to the start of a coexistence model with COVID-19 in November 2021. Some studies show insufficient evidence for public information campaigns to prevent and control COVID-19 (25). The reason for this is that public information campaigns are a subtle process that is integrated into the compliance of other policies and public awareness. In COVID-19, public information campaigns were a link between the country’s mind and the population, and to strengthen confidence in the fight against the epidemic. In addition, public information campaigns also assumed the role of COVID-19 vaccination promotion.

### 4.2 Testing policy

Testing policy is the key policy of COVID-19, the basis of contact tracing and isolation policy, the key link to breaking the chain of COVID-19 transmission, and an important source of data to evaluate a country’s COVID-19 prevention and control policy (26). The testing policy depends on a country’s medical and human resources, as well as testing capacity. In COVID-19, testing patterns, testing prices, and so on are reflected.

China continues to improve testing capacity and reduce testing prices. And use the advantages of traditional Chinese medicine to reduce the symptoms of patients, shorten the course of disease and reduce the conversion rate of severe diseases to save medical resources. In cluster epidemics, multiple rounds of mass testing are started locally, using grouped testing to conserve testing resources. Test results are presented together in trip codes. Japan initially allowed only symptomatic individuals to take tests, but later shifted to testing based on physician judgment. After Japan continued to improve its testing capabilities, the testing strategy was changed so that asymptomatic infected individuals could be tested for free (27). During the lockdown period, the Mongolian government initiated a mass testing campaign, the so-called “One door-one test,” which at least one member of each household was tested (28). South Korea established a car-free testing site to facilitate testing for citizens. Post-approval of antigen testing as a positive indicator.

In the face of COVID-19 and the shortage of healthcare resources, four countries have taken measures to conserve medical resources. However, there are also some problems, large-scale testing tends to produce people gathering with the risk of cross-infection, which requires strengthening the popular testing science and personnel management, maintaining social distancing, and standardized sterilization training for medical staff to reduce the risk of cross-infection. Omicron is hidden and difficult to detect, requiring multiple rounds of testing (29, 30). This poses a new dilemma for both the sustainability of health systems and health resource allocation. Countries should strengthen their testing capacity, rationalize the allocation of health resources, and enhance the early detection and identification of symptomatic populations in the early stages.

### 4.3 Contact tracing

Both contact tracing and testing policies are well-known public health intervention tools that have the least impact on the lives of non-potentially infected individuals (25) and combined with operational data can develop an early warning system for COVID-19 hotspots, predicting the location of potential outbreaks (31). Contact tracing is a key step in the epidemiological investigation and one of the most important measures in containment strategies. Its purpose is to cut off the transmission route of the new crown transmission and reverse the basic aspects of finding the source of infection and containment transmission.

Chinese Center for Disease Control and Prevention at all levels is the main focus of contact tracing in China. China insists on early detection and early reporting in contact tracing. After confirming the trajectories of confirmed patients and close contacts’ movements, they are communicated to the whole society. To speed up the tracking and save health resources, the form of place code combined with health code is adopted to facilitate the identification of temporal and spatial closeness. Japan focuses on large events, and cluster epidemics, and relies on a large network of local health officials. They consider confined spaces, crowded places, and close contact environments as the main risk factors that may lead to the occurrence of clustered cases, and focus on identifying clustered cases and eliminating widespread transmission, rather than trying to track each case. In Mongolia, close contacts are identified through video, interview, and interrogation, and scanning QR codes in public places are also used (16). However health workers are overburdened, there is burnout with contact tracing, and public distrust of the health care system (24). South Korea uses technology to send alerts to people within 100 meters of the area involved in epidemics and advises them to take testing. The South Korean government publishes the whereabouts of confirmed cases on its official website to make it easier for residents to inquire about close contacts.

Contact tracing is labor-intensive and a resource-intensive, multi-step process, making current contact tracing suboptimal (32, 33). The incubation period and asymptomatic infections pose a challenge for contact tracing. In the face of human resource shortages, four countries have adopted some electronic information technology tools to assist in contact tracing, but the study realizes that their overall utilization is not high (34). There are many other issues to be noted, First, the difficulty of omicron detection, the need to extend the length of storage of electronic information systems to ensure access to contact information; Second, the protection of public privacy, contact tracing involves a large number of public personal information, strict prevention of data leakage and serious violations of digital privacy events; Third, the smartphone penetration rate of the older adult groups is not high, the existing electronic information technology tools for the protection of the older adult groups is low (34, 35). In addition, Japan’s focus on identifying clustered cases in contact tracing is worthy of being handled by Western countries in cluster epidemics. Moreover, countries need to pay attention to the demands of medical personnel, protect the rights and interests of frontline medical workers, and motivate them to participate in epidemic prevention efforts.

### 4.4 Vaccination

Vaccines are considered a key response to COVID-19 and the most effective tool to combat the spread of COVID-19, and several studies have shown that vaccines can prevent severe illness and death (36). Herd or community immunity occurs when most of the population in a community is naturally or artificially immune (37). Vaccination is the most effective strategy to ensure a robust and durable immune response to SARS-CoV-2 virus and its variants. Global vaccination has been accelerated since COVID-19 vaccination started in 2021.

Four countries have vaccination rates above the world average. China began a mass vaccination campaign in January 2021 and gradually expanded to people over 60 years of age and children. Japan lags far behind other developed countries in vaccination, due to the lack of domestically produced vaccines and its reliance on imports, and the strict approval process for Japanese vaccines. Mongolia has strengthened international cooperation and extensive vaccination campaigns with the World Bank, UNICEF, etc., and has achieved high vaccination rates among less developed countries (24). At the request of South Korean government, many private clinics have conducted COVID-19 testing and vaccinations to make vaccinations easily available. High vaccination rates in South Korea also played an important role in the third or fourth wave (38).

Despite favorable results and promising outcomes, vaccine efficacy or effectiveness decreases over time and the duration of immunity may be short-lived (39, 40). Increased transmissibility and infectivity, severity of associated diseases, and virus mutations can all affect vaccine effectiveness (41). Omicron reinfection rates were much higher in fully vaccinated or previously infected individuals than in earlier variant strains, and all COVID-19 vaccines were significantly less effective against omicron variants (36, 42). The emergence of breakthrough infections and mutant strains will be a great challenge for the COVID-19 vaccine against COVID-19 (43, 44). While stepping up vaccination efforts, we should recognize that vaccines alone will not get any country out of this crisis and that a broad range of protective interventions is needed for the evolving pandemic (45).

### 4.5 Isolation and quarantine policy

Before vaccination, Isolation policy, social distancing, blockade, isolation of confirmed cases, and isolation of their contacts are considered important measures to prevent and control COVID-19 (46). Studies showed that isolating confirmed or suspected cases prevented 44 to 81% of cases and 31 to 63% of deaths (47). Due to differences in healthcare capacity, countries have adopted different admission strategies. Countries with containment and mitigation strategies show very significant differences in the use of isolation policies.

China adhered to the strategy of all those who indeed are isolated. It imposed a 76-day-long closure of Wuhan and built a square cabin hospital as a temporary hospital to centralize the treatment of mildly ill patients. Localities also followed isolation requirements by building isolation hotels and adopting 14-day centralized isolation, which was later shortened to 7 days. Japan promotes the allocation of medical resources by adopting self-isolation at home for mildly ill patients and medical institutions only accepting seriously ill patients. Mongolia has taken several initiatives to lockdown countries and cities for isolation, accompanied by active infection surveillance and self-isolation recommendations. South Korea designed and implemented community treatment centers to ensure the safety of non-COVID-19 patients by segregating areas for respiratory and non-respiratory patients.

Isolation and quarantine policy is aimed at incoming persons and close contacts and is an important part of the control of incoming persons and a gateway to avoid the virus inboard. The implementation of mass isolation or systematic social isolation allows individuals to directly eliminate the possibility of infection due to low physical contact (48). Japan, Mongolia, and South Korea have relaxed or lifted the quarantine of incoming persons, which poses a great challenge to prevent and control COVID-19. In addition, some studies have concluded that Isolation and quarantine policy measures harm the mental health and economic and social development of the population, especially on vulnerable groups and those with poor housing conditions (49, 50). In this case, the family quarantine model helps to ease tensions and for countries to be more precise in judging quarantine and blockade events to ensure normal working life order while safeguarding the prevention and control COVID-19.

In addition, when dealing with pandemics, countries should give full play to their own characteristics and advantages, such as China’s advantages in using traditional Chinese medicine to alleviate symptoms, prevent disease deterioration and enhance people’s immunity, to save strained medical resources. Japan takes advantage of its people’s high health literacy; And Vietnam are participating in COVID-19 prevention and control through grassroots cross-sectoral organizations.

A limitation of our study is that new cases per million are used in this study to describe the epidemic trend, but different countries have different detection capabilities and reporting standards, resulting in a certain degree of error. And the data limitations make it difficult to retrieve COVID-19 data and core health system measures for North Korea. Another limitation is that health system measures. More attention is paid to the release of measures, and less consideration is given to the actual use of measures.

In future research, study looks at optimizing data collection and processing methods, as well as exploring in depth the practical application of health system measures, with a view to providing more effective policy recommendations and decision support for pandemic response. Firstly, adding control variables or correct data errors caused by differences in reporting standards; secondly, designing a system of indicators for evaluating the response of health system to epidemic and quantifying measures; in addition, collecting more detailed data through qualitative research methods, such as in-depth interviews or case studies.

## 5 Conclusion

In containment strategies adopted by China and Mongolia, and mitigation strategies adopted by Japan and South Korea, health systems have played an important role in COVID-19 prevention and control. While promoting vaccination, countries should pay attention to non-pharmaceutical health system measures, as evidenced by: focusing on public information campaigns to lead public minds; strengthening detection capabilities for early detection and identification; using technical means to participate in contact tracing, and promoting precise judging isolation.

## Data availability statement

The original contributions presented in the study are included in the article/supplementary material, further inquiries can be directed to the corresponding author.

## Author contributions

JJ: Writing – original draft. WC: Funding acquisition, Project administration, Writing – review & editing.

## Footnotes

^1^ https://coronavirus.jhu.edu/?from=groupmessage

^2^ https://covid19.who.int/table

^3^ http://www.nhc.gov.cn

^4^ https://www.mhlw.go.jp/index.html

^5^ https://moh.gov.mn/

^6^ https://www.mohw.go.kr/eng/index.jsp

^7^ http://en.nhc.gov.cn/index.html

^8^ http://www.mw.go.kr/

## References

[1] Cucinotta D Vanelli M. WHO declares COVID-19 a pandemic. Acta Biomed. (2020) 91:157–60. doi: 10.23750/abm.v91i1.9397, PMID: 32191675 PMC7569573

[2] Population Data Portal. Available at: https://pdp.unfpa.org/?_ga=2.29710518.865667680.1662344878-1467124044.1662344878 (Accessed September 5, 2022).

[3] Classification of omicron (B.1.1.529): SARS-CoV-2 variant of concern. Available at: https://www.who.int/news/item/26-11-2021-classification-of-omicron-(b.1.1.529)-sars-cov-2-variant-of-concern (Accessed April 13, 2022).

[4] Meo SA Meo AS Al-Jassir FF Klonoff DC. Omicron SARS-CoV-2 new variant: global prevalence and biological and clinical characteristics. Eur Rev Med Pharmacol Sci. (2021) 25:8012–8. doi: 10.26355/eurrev_202112_27652, PMID: 34982465

[5] Kwok KO Lai F Wei WI Wong SYS Tang JWT. Herd immunity – estimating the level required to halt the COVID-19 epidemics in affected countries. J Infect. (2020) 80:e32–3. doi: 10.1016/j.jinf.2020.03.027, PMID: 32209383 PMC7151357

[6] Tham TY Tran TL Prueksaritanond S Isidro JS Setia S Welluppillai V. Integrated health care systems in Asia: an urgent necessity. Clin Interv Aging. (2018) 13:2527–38. doi: 10.2147/CIA.S185048, PMID: 30587945 PMC6298881

[7] Japan health policy NOW – 4.1 overview of Japan’s healthcare delivery system. Available at: http://japanhpn.org/en/section-4-1/ (Accessed May 1, 2024).

[8] Ritchie H Mathieu E Rodés-Guirao L Appel C Giattino C Ortiz-Ospina E. Coronavirus pandemic (COVID-19). Our World in Data (2020). Available at: https://ourworldindata.org/covid-vaccinations (Accessed September 5, 2022).

[9] Kadkhoda K. Herd immunity to COVID-19. Am J Clin Pathol. (2021) 155:471–2. doi: 10.1093/ajcp/aqaa272, PMID: 33399182 PMC7929447

[10] Long COVID: let patients help define long-lasting COVID symptoms. Nature. (2020) 586:170–11. doi: 10.1038/d41586-020-02796-2, PMID: 33029005

[11] Ledford H. Coronavirus reinfections: three questions scientists are asking. Nature. (2020) 585:168–9. doi: 10.1038/d41586-020-02506-y, PMID: 32887957

[12] Parry J. Covid-19: Hong Kong scientists report first confirmed case of reinfection. BMJ. (2020) 370:m3340. doi: 10.1136/bmj.m3340, PMID: 32847834

[13] Vadlamannati KC Cooray A de Soysa I. Health-system equity, egalitarian democracy and COVID-19 outcomes: an empirical analysis. Scand J Public Health. (2021) 49:104–13. doi: 10.1177/1403494820982106 33427079 PMC7797351

[14] Chang AY Cullen MR Harrington RA Barry M. The impact of novel coronavirus COVID-19 on non-communicable disease patients and health systems: a review. J Intern Med. (2021) 289:450–62. doi: 10.1111/joim.13184, PMID: 33020988 PMC7675448

[15] Lai J Ma S Wang Y Cai Z Hu J Wei N. Factors associated with mental health outcomes among health care workers exposed to coronavirus disease 2019. JAMA Netw Open. (2020) 3:e203976. doi: 10.1001/jamanetworkopen.2020.3976, PMID: 32202646 PMC7090843

[16] Bayasgalan T Anuurad E Byambaa E. COVID-19 and public health efforts in Mongolia: a lesson maybe learned? J Clin Transl Sci. (2021) 5:e18. doi: 10.1017/cts.2020.510

[17] Alfano V Ercolano S. The efficacy of lockdown against COVID-19: a cross-country panel analysis. Appl Health Econ Health Policy. (2020) 18:509–17. doi: 10.1007/s40258-020-00596-3, PMID: 32495067 PMC7268966

[18] Weiss JA Tschirhart M. Public information campaigns as policy instruments. J Policy Anal Manage. (1994) 13:82–119. doi: 10.2307/3325092

[19] Solovei A Van Den PB. The effects of five public information campaigns: the role of interpersonal communication. Communications. (2020) 45:586–602. doi: 10.1515/commun-2020-2089

[20] Podolsky MI Present I Neumann PJ Kim DD. A systematic review of economic evaluations of COVID-19 interventions: considerations of non-health impacts and distributional issues. Value Health. (2022) 25:1298–306. doi: 10.1016/j.jval.2022.02.003, PMID: 35398012 PMC8986127

[21] Lazarus JV Ratzan S Palayew A Billari FC Binagwaho A Kimball S. COVID-SCORE: a global survey to assess public perceptions of government responses to COVID-19 (COVID-SCORE-10). PLoS One. (2020) 15:e0240011. doi: 10.1371/journal.pone.0240011, PMID: 33022023 PMC7538106

[22] Takahashi H Terada I Higuchi T Takada D Shin J-H Kunisawa S. The relationship between new PCR positive cases and going out in public during the COVID-19 epidemic in Japan. PLoS One. (2022) 17:e0266342. doi: 10.1371/journal.pone.0266342, PMID: 35617292 PMC9135210

[23] Konishi T. Effect of control measures on the pattern of COVID-19 epidemics in Japan. PeerJ. (2021) 9:e12215. doi: 10.7717/peerj.12215, PMID: 34692252 PMC8483016

[24] Dagvadorj A Jantsansengee B Balogun OO Baasankhuu T Lkhagvaa B. Health emergency preparedness and response to the COVID-19 pandemic: lessons learnt from Mongolia. Lancet Reg Health West Pac. (2022) 21:100436. doi: 10.1016/j.lanwpc.2022.100436, PMID: 35350463 PMC8948500

[25] Liu Y Morgenstern C Kelly J Lowe R Munday J Villabona-Arenas CJ. The impact of non-pharmaceutical interventions on SARS-CoV-2 transmission across 130 countries and territories. BMC Med. (2021) 19:40. doi: 10.1186/s12916-020-01872-8, PMID: 33541353 PMC7861967

[26] Kuster AC Overgaard HJ. A novel comprehensive metric to assess effectiveness of COVID-19 testing: inter-country comparison and association with geography, government, and policy response. PLoS One. (2021) 16:e0248176. doi: 10.1371/journal.pone.0248176, PMID: 33667280 PMC7935311

[27] Kurihara M Kamata K Nakahara S Kitazawa K Koizumi S Tokuda Y. Healthcare use and RT-PCR testing during the first wave of the COVID-19 pandemic in Japan. J Gen Fam Med. (2021) 23:3–8. doi: 10.1002/jgf2.512, PMID: 35004104 PMC8721334

[28] Dorjdagva J Batbaatar E Kauhanen J. Mass testing for COVID-19 in Ulaanbaatar, Mongolia: “one door-one test” approach. Lancet Reg Health West Pac. (2021) 9:100149. doi: 10.1016/j.lanwpc.2021.100149, PMID: 34327444 PMC8315464

[29] Ferré VM Peiffer-Smadja N Visseaux B Descamps D Ghosn J Charpentier C. Omicron SARS-CoV-2 variant: what we know and what we don’t. Anaesth Crit care. Pain Med. (2021) 41:100998. doi: 10.1016/j.accpm.2021.100998, PMID: 34902630 PMC8660660

[30] Chen J Wang R Gilby NB Wei G-W. Omicron variant (B.1.1.529): infectivity, vaccine breakthrough, and antibody resistance. J Chem Inf Model. (2022) 62:412–22. doi: 10.1021/acs.jcim.1c01451, PMID: 34989238 PMC8751645

[31] Yabe T Tsubouchi K Sekimoto Y Ukkusuri SV. Early warning of COVID-19 hotspots using human mobility and web search query data. Comput Environ Urban Syst. (2022) 92:101747. doi: 10.1016/j.compenvurbsys.2021.101747, PMID: 34931101 PMC8673829

[32] Lash RR Moonan PK Byers BL Bonacci RA Bonner KE Donahue M. COVID-19 case investigation and contact tracing in the US, 2020. JAMA Netw Open. (2021) 4:e2115850. doi: 10.1001/jamanetworkopen.2021.15850, PMID: 34081135 PMC8176334

[33] CDC. Health departments. Centers for Disease Control and Prevention (2020). Available at: https://www.cdc.gov/coronavirus/2019-ncov/php/contact-tracing/contact-tracing-plan/overview.html (Accessed September 13, 2022).

[34] He Y Yatsuya H Ota A Tabuchi T. The association of public trust with the utilization of digital contact tracing for COVID-19 in Japan. Public Health Pract. (2022) 4:100279. doi: 10.1016/j.puhip.2022.100279, PMID: 35719273 PMC9187877

[35] Abeler J Bäcker M Buermeyer U Zillessen H. COVID-19 contact tracing and data protection can go together. JMIR Mhealth Uhealth. (2020) 8:e19359. doi: 10.2196/19359, PMID: 32294052 PMC7173240

[36] Helmy SA El-Morsi RM Helmy SAM El-Masry SM. Towards novel nano-based vaccine platforms for SARS-CoV-2 and its variants of concern: advances, challenges and limitations. J Drug Deliv Sci Technol. (2022) 76:103762. doi: 10.1016/j.jddst.2022.103762, PMID: 36097606 PMC9452404

[37] Neagu M. The bumpy road to achieve herd immunity in COVID-19. J Immunoass Immunochem. (2020) 41:928–45. doi: 10.1080/15321819.2020.1833919, PMID: 33086932

[38] Jeon J Han C Kim T Lee S. Evolution of responses to COVID-19 and epidemiological characteristics in South Korea. Int J Environ Res Public Health. (2022) 19:4056. doi: 10.3390/ijerph19074056, PMID: 35409740 PMC8997838

[39] Feikin DR Higdon MM Abu-Raddad LJ Andrews N Araos R Goldberg Y. Duration of effectiveness of vaccines against SARS-CoV-2 infection and COVID-19 disease: results of a systematic review and meta-regression. Lancet. (2022) 399:924–44. doi: 10.1016/S0140-6736(22)00152-0, PMID: 35202601 PMC8863502

[40] Anderson RM Vegvari C Truscott J Collyer BS. Challenges in creating herd immunity to SARS-CoV-2 infection by mass vaccination. Lancet. (2020) 396:1614–6. doi: 10.1016/S0140-6736(20)32318-7, PMID: 33159850 PMC7836302

[41] Deng X Garcia-Knight MA Khalid MM Servellita V Wang C Morris MK. Transmission, infectivity, and neutralization of a spike L452R SARS-CoV-2 variant. Cell. (2021) 184:3426–3437.e8. doi: 10.1016/j.cell.2021.04.025 33991487 PMC8057738

[42] Mahase E. Covid-19: omicron and the need for boosters. BMJ. (2021) 375:n3079. doi: 10.1136/bmj.n3079 34906956

[43] Ferraz MVF Moreira EG Coêlho DF Wallau GL Lins RD. Immune evasion of SARS-CoV-2 variants of concern is driven by low affinity to neutralizing antibodies. Chem Commun. (2021) 57:6094–7. doi: 10.1039/D1CC01747K, PMID: 34037640

[44] Garcia-Beltran WF Lam EC St. Denis K Nitido AD Garcia ZH Hauser BM. Multiple SARS-CoV-2 variants escape neutralization by vaccine-induced humoral immunity. Cell. (2021) 184:2372–2383.e9. doi: 10.1016/j.cell.2021.03.013, PMID: 33743213 PMC7953441

[45] WHO director-General’s opening remarks at the media briefing on COVID-19 - 14 (2021). Available at: https://www.who.int/director-general/speeches/detail/who-director-general-s-opening-remarks-at-the-media-briefing-on-covid-19---14-december-2021 (Accessed April 11, 2022).

[46] Lau H Khosrawipour V Kocbach P Mikolajczyk A Schubert J Bania J. The positive impact of lockdown in Wuhan on containing the COVID-19 outbreak in China. J Travel Med. (2020) 27:taaa037. doi: 10.1093/jtm/taaa037, PMID: 32181488 PMC7184469

[47] Nussbaumer-Streit B Mayr V Dobrescu AI Chapman A Persad E Klerings I. Quarantine alone or in combination with other public health measures to control COVID-19: a rapid review. Cochrane Database Syst Rev. (2020) 4:CD013574. doi: 10.1002/14651858.CD013574 32267544 PMC7141753

[48] Utsumi S Arefin MR Tatsukawa Y Tanimoto J. How and to what extent does the anti-social behavior of violating self-quarantine measures increase the spread of disease? Chaos, Solitons Fractals. (2022) 159:112178. doi: 10.1016/j.chaos.2022.112178, PMID: 35578625 PMC9094739

[49] Haesebaert F Haesebaert J Zante E Franck N. Who maintains good mental health in a locked-down country? A French nationwide online survey of 11,391 participants. Health Place. (2020) 66:102440. doi: 10.1016/j.healthplace.2020.102440, PMID: 32947185 PMC7490637

[50] Gloster AT Lamnisos D Lubenko J Presti G Squatrito V Constantinou M. Impact of COVID-19 pandemic on mental health: an international study. PLoS One. (2020) 15:e0244809. doi: 10.1371/journal.pone.0244809, PMID: 33382859 PMC7774914

